# The *Drosophila* FoxA Ortholog Fork Head Regulates Growth and Gene Expression Downstream of Target of Rapamycin

**DOI:** 10.1371/journal.pone.0015171

**Published:** 2010-12-31

**Authors:** Margret H. Bülow, Ruedi Aebersold, Michael J. Pankratz, Martin A. Jünger

**Affiliations:** 1 Institute of Molecular Systems Biology, Swiss Federal Institute of Technology (ETH) Zurich, Zurich, Switzerland; 2 Department of Molecular Brain Physiology and Behavior, Life and Medical Sciences (LIMES) Institute, University of Bonn, Bonn, Germany; 3 Competence Center for Systems Physiology and Metabolic Diseases (CC-SPMD), Swiss Federal Institute of Technology (ETH) Zurich, Zurich, Switzerland; 4 Faculty of Science, University of Zurich, Zurich, Switzerland; University of Minnesota, United States of America

## Abstract

Forkhead transcription factors of the FoxO subfamily regulate gene expression programs downstream of the insulin signaling network. It is less clear which proteins mediate transcriptional control exerted by Target of rapamycin (TOR) signaling, but recent studies in nematodes suggest a role for FoxA transcription factors downstream of TOR. In this study we present evidence that outlines a similar connection in *Drosophila*, in which the FoxA protein Fork head (FKH) regulates cellular and organismal size downstream of TOR. We find that ectopic expression and targeted knockdown of FKH in larval tissues elicits different size phenotypes depending on nutrient state and TOR signaling levels. FKH overexpression has a negative effect on growth under fed conditions, and this phenotype is not further exacerbated by inhibition of TOR via rapamycin feeding. Under conditions of starvation or low TOR signaling levels, knockdown of FKH attenuates the size reduction associated with these conditions. Subcellular localization of endogenous FKH protein is shifted from predominantly cytoplasmic on a high-protein diet to a pronounced nuclear accumulation in animals with reduced levels of TOR or fed with rapamycin. Two putative FKH target genes, *CG6770* and *cabut*, are transcriptionally induced by rapamycin or FKH expression, and silenced by FKH knockdown. Induction of both target genes in heterozygous *TOR* mutant animals is suppressed by mutations in *fkh*. Furthermore, TOR signaling levels and FKH impact on transcription of the dFOXO target gene *d4E-BP*, implying a point of crosstalk with the insulin pathway. In summary, our observations show that an alteration of FKH levels has an effect on cellular and organismal size, and that FKH function is required for the growth inhibition and target gene induction caused by low TOR signaling levels.

## Introduction

Transcription factors belonging to the winged helix/*forkhead* box (Fox) family are implicated in a variety of biological processes ranging from embryonic development to the regulation of metabolism, growth, cell death and organismal lifespan [1]. Members of this transcription factor family share a conserved 110-residue DNA binding domain first discovered in the *Drosophila* Fork Head protein, which therefore and because of structural reasons is referred to as the forkhead or winged helix domain. The family is divided into subclasses labeled with the letters A to S, and this categorization is based on amino acid sequence similarity in the forkhead domain [2]. For most Fox proteins, knowledge is scarce about how they are interfaced with upstream signaling pathways. A well characterized group is the FoxO subfamily which, among input from other pathways, is regulated by the insulin signaling module in a way that is conserved between the nematode *Caenorhabditis elegans* and humans. This involves direct phosphorylation by insulin-induced kinases, binding to 14-3-3 proteins and nucleocytoplasmic shuttling [3]. The insulin-forkhead connection was first described in *C. elegans*, where mutations in the insulin receptor gene *daf-2* are completely suppressed by mutations in the FoxO transcription factor gene *daf-16* [4], [5]. Members of the FoxA subfamily are also important players in metabolism and regulated by insulin, but whether the exact mechanism also involves nuclear exclusion is still a matter of debate and less clear than for FoxO proteins [6]. Once again, pioneering research in *C. elegans* started to uncover a link between a growth control pathway and a forkhead transcription factor, this time between the worm Target of rapamycin (TOR) homolog LET-363 and the FoxA protein PHA-4. The first study in this line of evidence described the longevity of worms with reduced levels of TOR signaling. In contrast to the *daf-16*-dependent lifespan increase of insulin receptor mutant worms, long life conferred by low levels of TOR is not affected by *daf-16* mutations [7]. This suggested that insulin and TOR signaling regulate lifespan through distinct downstream transcriptional regulators. Another condition which, similar to reduced TOR signaling, can prolong life in nematodes in a DAF-16-independent fashion is dietary restriction. LET-363 is involved in the dietary restriction response induced by mutations in either *eat-2* which lead to reduced food intake by impaired pharyngeal pumping [8], or in *pep-2* mutants which display compromised intestinal uptake of dietary peptides [9]. PHA-4 was recently identified as the forkhead transcription factor which is necessary to increase lifespan under multiple conditions of dietary restriction, such as lowering the concentration of bacteria fed to the worms in culture or *eat-2* mutations, but not under conditions of lowered insulin signaling [10], making it a candidate for a transcriptional effector downstream of TOR signaling. This working hypothesis was confirmed by the finding that PHA4 is required for the lifespan extension elicited by reduced LET-363/TOR or RSKS-1/S6 kinase levels, both of which are independent of DAF-16 function [11]. These observations prompted us to investigate a possible link between TOR signaling and the transcription factor Fork head (FKH), which among the 18 *Drosophila* forkhead proteins is the only one belonging to the FoxA subfamily [12]. Here we present evidence that in addition to its established role in embryonic development and regulation of salivary gland cell death in the larva, FKH controls cell and organismal size, that its subcellular localization is regulated by TOR signaling and that it is necessary for the expression of rapamycin- and starvation-responsive genes as well as for rapamycin-induced inhibition of growth. For the first time in *Drosophila*, we describe an interaction between TOR and a FoxA protein, which is in agreement with the observations made in *C. elegans*. In addition, our findings yield novel insights about the regulation of growth by FKH, and how FKH and dFOXO are partially redundant in the regulation of *d4E-BP* expression.

## Results

### Alteration of FKH levels elicits organism and cell size phenotypes which are sensitive to nutrient conditions and TOR signaling

**Figure 1:**
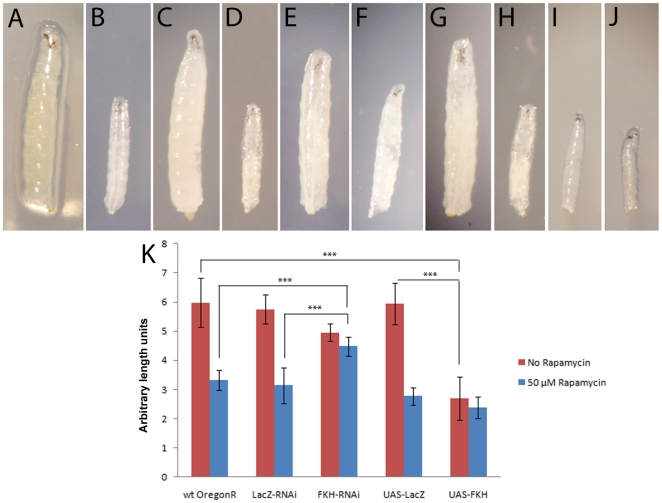
FKH levels influence larval body size. Pictures show 72 h old larvae (±2h) which were treated at 24 h after egg deposition (AED) with 0 (**A**, **C**, **E**, **G**, **I**) or 50 µM (**B**, **D**, **F**, **H**, **J**) rapamycin for 48 h. A, B: OregonR wildtype control. C, D: *y w UAS-LacZ-RNAi*; *arm-Gal4/+*. Treating control larvae with 50 µM rapamycin leads to severe reduction of growth. E, F: *w; arm-Gal4/+*; *UAS-FKH-RNAi/+*. Untreated larvae are slightly smaller than controls, but size reduction by rapamycin feeding is less pronounced in larvae with low FKH levels. G, H: w;; *ppl-Gal4/UAS-LacZ*. I, J: *w;; ppl-Gal4/UAS-FKH*. Overexpression of FKH leads to a severe reduction in body size similar to that resulting from rapamycin feeding. At 25°C, overexpression furthermore leads to larval lethality in the 2^nd^ instar. Rapamycin feeding of FKH-overexpressing larvae has almost no additive effect to the reduction in body size. (**K**) Quantitation of larval body size shows that rapamycin-treated larvae with low FKH levels are significantly larger than control larvae and that untreated larvae with high FKH levels are significantly smaller than control larvae. Significance was tested using an unpaired 2-tailed Student's t-test. *** = p<0.001. Error bars represent standard error of the mean (SEM).

As in other organisms, TOR signaling in *Drosophila* is involved in the nutrient-dependent regulation of cellular and organismal growth [13], [14]. We therefore first asked whether an alteration of FKH levels *in vivo* would have an effect on growth. As homozygous *fkh* mutants are embryonic lethal [15], we applied the GAL4/UAS system to either knock down FKH in larval tissues by RNA interference or to raise FKH levels by ectopic overexpression, each under different nutrient conditions. As shown in Figure 1, overexpression of FKH in the larval fatbody induced by the *pumpless* driver causes a severe reduction in body size (Figure 1I and K), similar to the one resulting from TOR inhibition induced by rearing the larvae on rapamycin-containing food (Figure 1B). Rapamycin does not significantly further reduce the size of FKH-overexpressing animals (Figure 1J). Moderate ubiquitous RNAi knockdown of FKH with the *armadillo* driver leads to a slight decrease in larval size under fed conditions (Figure 1E). Rapamycin feeding causes a reduction in larval size via inhibition of TOR signaling. However, this phenotype is less strong in animals with repressed FKH levels (Figure 1F and K), suggesting that FKH is required for rapamycin-induced growth inhibition. Expression of a negative control inverted repeat directed against lacZ had no significant impact on larval size under both conditions (Figure 1C, D and K), ruling out unspecific effects of double-stranded RNA expression. Likewise, ectopic expression of β-galactosidase in UAS-lacZ control animals had no effect on larval size (Figure 1G, H and K). Quantitative realtime PCR was performed to measure the efficiency of *fkh* knockdown by RNAi in this experimental setup, and *fkh* transcript levels were found to be reduced by 60% compared to wildtype larvae (Figure S1).

**Figure 2:**
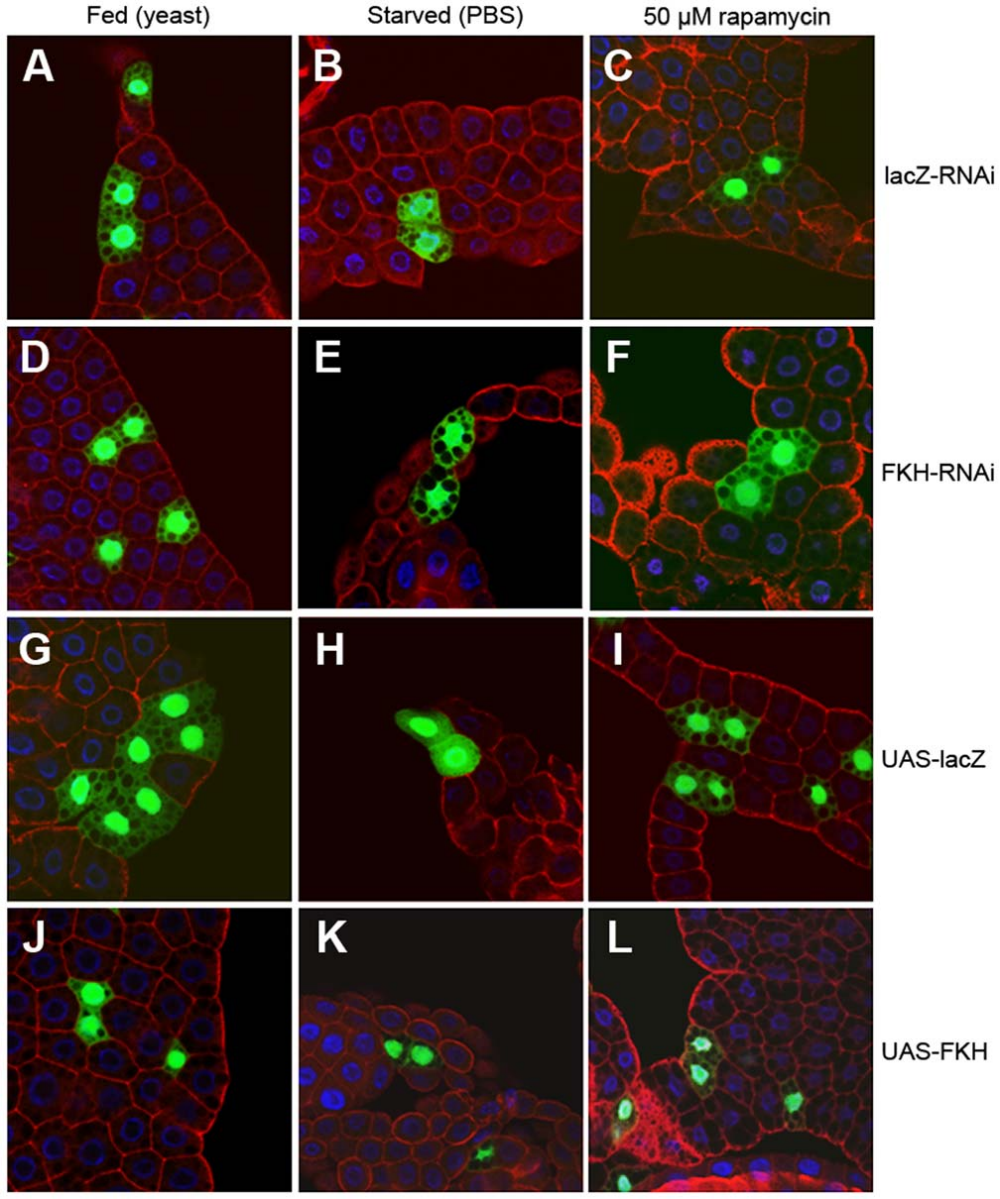
FKH levels influence cell size in the larval fatbody. Pictures show confocal sections of larval fatbodies, using the fly line *y w hs-flp;; Act>CD2>Gal4 UAS-GFP* to drive *UAS-FKH-RNAi*, overexpression and control responder lines. Cells expressing the transgene are marked by the co-expression of GFP, whereas the non-fluorescent serve as wild-type controls within the same tissue sample. Larvae were reared on yeast for 64 h AED, or starved on PBS for 24 h after growing on yeast for 64 h, or treated with rapamycin for 24 h after growing on yeast for 48 h. Tissue was stained with α-GFP (green), α-CD2 (red) and DAPI (blue). (**A**–**C**, **G**–**I**) Controls show no obvious phenotype in the GFP-positive cells. (**D**–**F**) Cells expressing FKH RNAi have no growth phenotype under fed conditions, but are larger than the surrounding tissue in starved and rapamycin-treated larvae. (**J**–**L**) Cells overexpressing FKH are smaller than the surrounding tissue under fed conditions.

**Figure 3:**
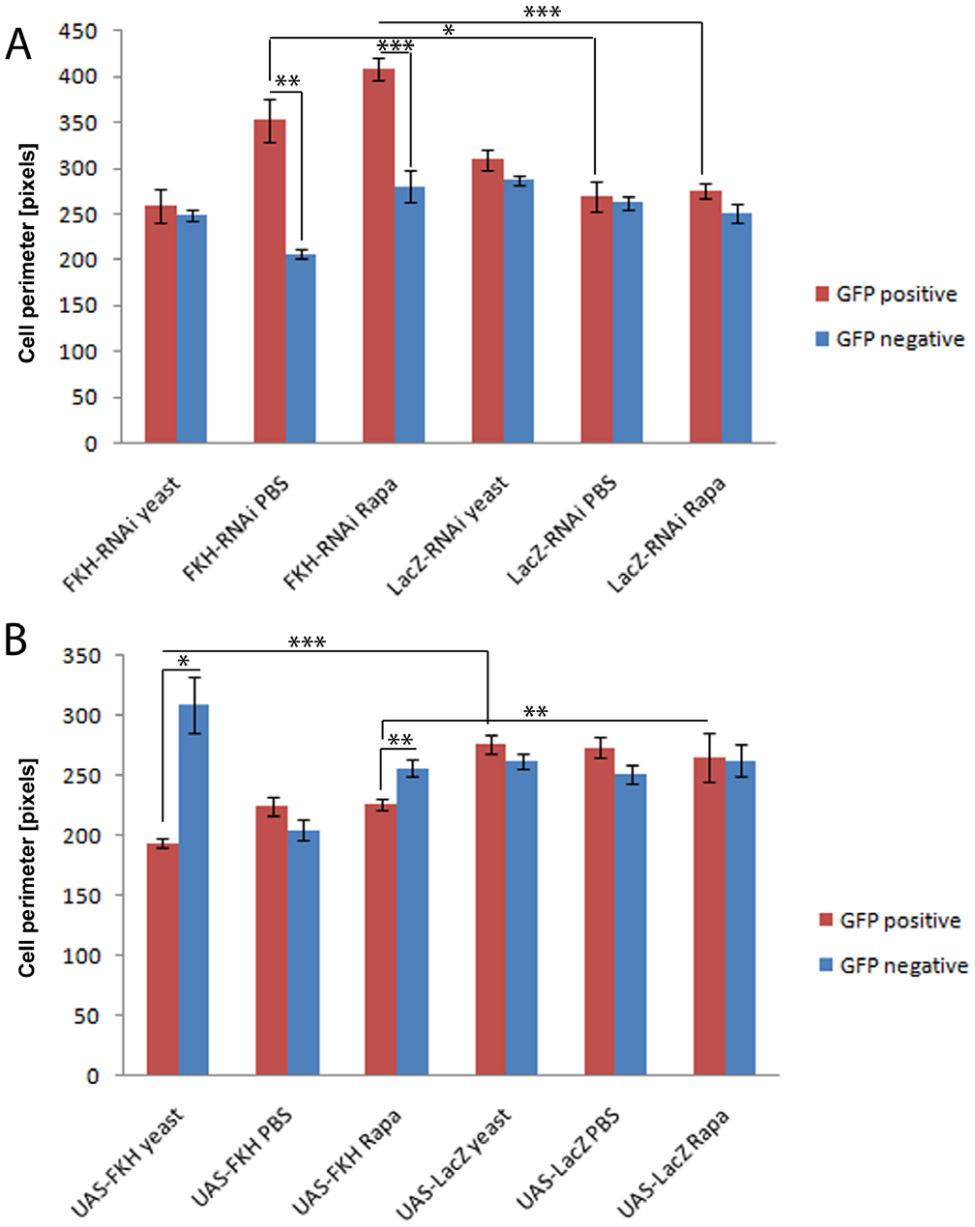
Quantitation of the cell size phenotypes shown in figure 2. Cell size was analyzed by measuring the cell perimeter with the software ImageJ. Blue bars represent the GFP-negative wild-type cells, red bars represent the cells expressing the transgene. Larvae were fed with yeast paste, starved on PBS or treated with 50 µM rapamycin. (**A**) Effect of FKH knockdown on cell size. Larvae were of the genotype *y w hs-flp;; Act>CD2>Gal4 UAS-GFP/UAS-FKH-RNAi* (bars labeled “FKH-RNAi”) or *y w hs-flp;; Act>CD2>Gal4 UAS-GFP/UAS-LacZ-RNAi* (bars labeled “LacZ-RNAi”) as an unspecific control. Cells expressing FKH dsRNA are significantly larger than the surrounding tissue in larvae which were starved on PBS or treated with rapamycin. (**B**) Effect of FKH overexpression on cell size. Larvae were of the genotype *y w hs-flp;; Act>CD2>Gal4 UAS-GFP/UAS-FKH* (bars labeled “UAS-FKH”) or *y w hs-flp;; Act>CD2>Gal4 UAS-GFP/UAS-LacZ* (bars labeled “UAS-LacZ”) as an unspecific control. Cells which overexpress FKH are significantly smaller than the surrounding tissue in fed larvae, but not in larvae starved on PBS. They are also significantly smaller than wild-type cells in larvae treated with rapamycin. Significance was tested using an unpaired 2-tailed Student's t-test. * = p<0.05; ** = p<0.01; *** = p<0.001. Error bars represent SEM. Because the larvae are not age-matched across the different conditions (see Materials and Methods), we base our statements on the comparison of transgene-expressing cells to the wild-type control cells within the same sample, and not on the comparison of absolute cell sizes across different conditions.

In a second set of experiments, we analyzed the effect of FKH overexpression or knockdown on cell size. The ‘FLP-out’ system [16] was used to overexpress or silence FKH in cell clones in the larval fatbody. The effects on cell size were similar to those on organismal size and are displayed in Figure 2, the quantitative and statistical analysis of cell size phenotypes is shown in Figure 3. For each genotype and condition, we compared the size of transgene-expressing cells which are marked by the co-expression of nuclear green fluorescent protein (GFP) to the size of the non-fluorescent wild-type cells within the same tissue sample. Cells overexpressing FKH are significantly smaller than wild-type cells in fed animals (Figure 2J and 3B) but not in starved larvae (Figure 2K and 3B). In rapamycin-fed larvae, clonal FKH expression slightly reduced cell size, but to a much lesser extent compared to yeast-fed animals (Figure 2L and 3B). This indicates that cells are most susceptibe to growth inhibition by ectopic FKH expression under conditions of dietary protein abundance and active TOR signaling. Conversely, RNAi-induced silencing of FKH expression in cell clones had no significant effect on cell size in fatbodies of fed larvae (Figure 2D and 3A), but under conditions of starvation or rapamycin feeding, FKH knockdown elicited increased cell growth compared to control cells within the same tissue (Figure 2E, F and 3A). The observation that cells with low FKH levels are larger than the neighboring wild-type cells in tissues subjected to starvation or TOR inhibition supports the model that FKH is required for the inhibition of growth in response to these conditions. As in the larval body size experiments described above, fat body cell size was unaffected by clonal expression of the negative control constructs UAS-lacZ-RNAi (Figure 2D–F and 3A) or UAS-lacZ (Figure 2J–L and 3B). In this experimental setup, the FKH phenotypes observed correspond qualitatively to those of negatively regulating TOR signaling components. As shown in Figure S4, co-expression of TSC1 and TSC2 [17], negative upstream regulators of TOR, strongly reduces cell size in feeding larvae, while the size reduction is much less pronounced in starved animals. Conversely, expression of the small GTPase Rheb, which activates TOR signaling, leads to a mild cell size increase under conditions of nutrient abundance and to a stronger one under starvation, as has been reported previously [18], [19].

### Subcellular localization of FKH depends on nutrient and TOR signaling levels

**Figure 4:**
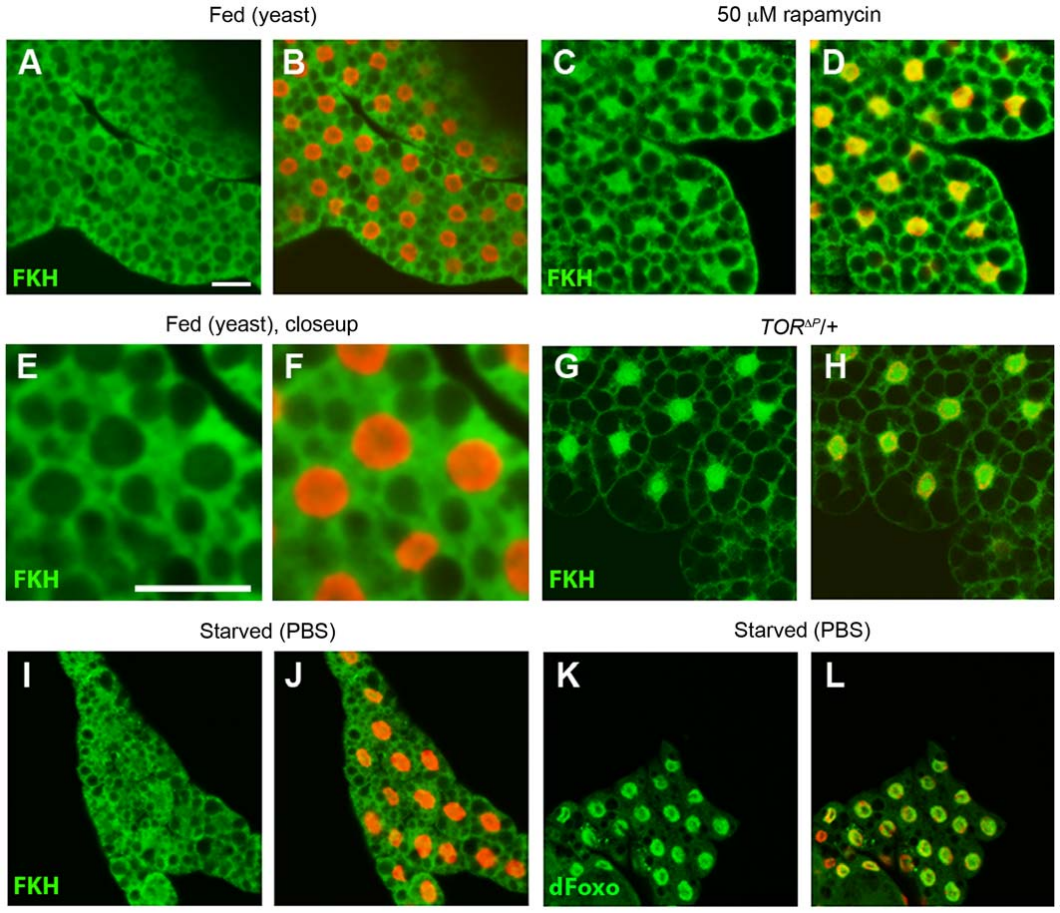
Subcellular localization of FKH is modulated by nutrient availability and TOR signaling. Confocal sections of larval fatbody are shown. For each section, one panel shows only the signal derived from the FKH or dFOXO antibody in green, and a second panel an additional nuclear counterstain with DAPI pseudo-colored in red. (**A**, **B**) FKH is excluded from the nucleus in fatbodies of wildtype 2^nd^ instar larvae fed on yeast. (**C**, **D**) FKH accumulates in the nucleus in fatbodies of wildtype larvae treated with 50 µM rapamycin. (**E**, **F**) Closeup of A, B. (**G**, **H**) FKH is nuclear in heterozygous *TOR* mutants (*y w; dTOR^ΔP^/+*). (**I**, **J**) Nuclear exclusion of FKH is not observed in larvae subjected to starvation on PBS. (**K**, **L**) In contrast to FKH, dFOXO shows a clear nuclear localization in wildtype larvae starved on PBS. Scale bar is 20 µm.

In the next step, we sought to investigate whether nutrient or TOR signaling levels have an influence on the subcellular localization of FKH protein. To monitor localization of the endogenous protein in tissues, we generated an affinity-purified polyclonal antibody against FKH. To ascertain the specificity of the new tool, western blot and immunohistochemical control experiments were performed which showed that the antibody could be used to detect endogenous as well as transgenically encoded FKH protein in larval extracts with very little unspecific background (Figure S2A). Furthermore, immunostainings visualized endogenous and overexpressed epitope-tagged FKH protein, and it could be observed that the main nuclear signal recognized by the antibody is indeed FKH (Figure S2B–E). Based on these control experiments, we then analyzed endogenous FKH localization in the larval fatbody under conditions of different nutrient and TOR signaling levels. When larvae were fed yeast paste, which contains a higher fraction of dietary proteins and amino acids compared to sugar-cornmeal-based fly food [20], [21], FKH was found to be localized in the cytoplasm of fatbody cells and almost completely excluded from the nuclei (Figure 4A, B and 4E, F). In larvae starved on PBS, nuclear exclusion was not observed, and the contours of the nuclei were not visible due to the absence of nuclear signal as in the tissue from animals reared on a high-protein diet (Figure 4I, J). Significant nuclear accumulation did not occur either, but the protein was rather evenly distributed within the cells. It should be noted that under this starvation condition, dFOXO shows a strong nuclear localization (Figure 4K, L). In contrast to the condition of complete starvation, in fatbodies from rapamycin-fed (Figure 4C, D) or heterozygous *TOR* mutant larvae (Figure 4G, H), FKH was localized predominantly nuclear, although a prominent cytoplasmic signal remained detectable. This suggests that TOR activity is required to keep FKH protein sequestered in the cytoplasm under conditions of dietary protein abundance. Importantly, this effect of TOR signaling on FKH localization was only observed on the endogenous protein. In contrast, overexpressed FKH was found to be constitutively nuclear in the larval fatbody (Figure S2B–E) as well as in cultured S2R+ cells (Figure S2F–K) even under conditions of nutrient abundance and high insulin/TOR signaling levels.

### The expression of *cabut* and *CG6770* is regulated by FKH and TOR signaling

**Figure 5:**
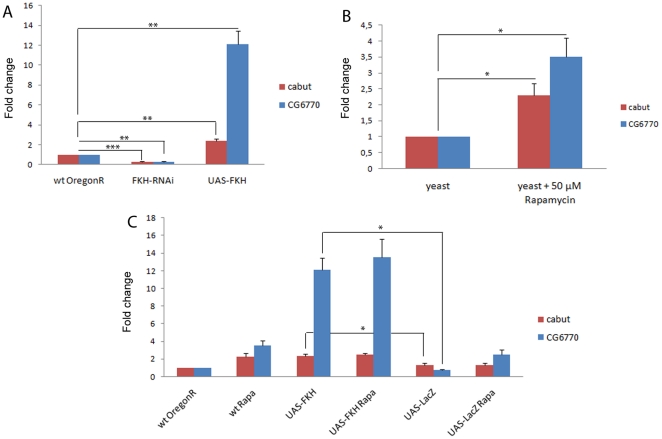
Transcription of *CG6770* and *cabut* is regulated by FKH and rapamycin. Realtime qPCR was performed to quantify mRNAs in larval extracts. (**A**) Compared to wildtype animals, the putative FKH targets *CG6770* and *cabut* are downregulated in yeast-fed larvae with low FKH levels (*w; arm-Gal4/+*; *UAS-FKH-RNAi/+*) and upregulated in larvae overexpressing FKH (*w;; ppl-Gal4/UAS-FKH*). (**B**) *CG6770* and *cabut* transcription is induced in wildtype larvae fed with 50 µM rapamycin. (**C**) *CG6770* and *cabut* mRNA levels are significantly elevated upon rapamycin treatment and in larvae with high FKH levels in comparison to the wildtype and unspecific controls (*w;; ppl-Gal4/UAS-LacZ*). Rapamycin treatment of larvae with high FKH levels has no significant additive effect on target gene expression. Significance was tested using an unpaired 2-tailed Student's t-test. * = p<0.05; ** = p<0.01; *** = p<0.001. Error bars represent SEM.

**Figure 6:**
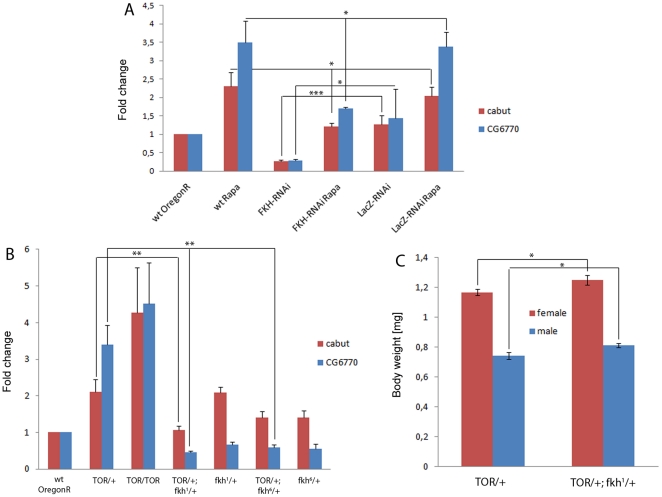
FKH is required for the response to rapamycin and lowered TOR levels. Realtime qPCR was performed to quantify mRNAs in larval extracts. (**A**) Transcription of *CG6770* and *cabut* is induced upon rapamycin treatment (Rapa) and downregulated in larvae with low FKH levels (*w; arm-Gal4/+*; *UAS-FKH-RNAi/+*). Compared to rapamycin-fed wildtype and unspecific control animals (*w; arm-Gal4/+*; *UAS-LacZ-RNAi/+*), expression of *CG6770* and *cabut* is significantly lower in rapamycin-fed FKH-RNAi larvae. (**B**) Consistent with the high expression of *CG6770* and *cabut* in rapamycin-treated larvae, these genes are transcriptionally upregulated in *dTOR* mutants (*y w; dTOR^ΔP^/+ and y w*; *dTOR^ΔP^/dTOR^ΔP^*). The elevated expression is suppressed in larvae transheterozygous for dTOR and FKH (*y w; dTOR^ΔP^/+*; *fkh^1^/+ and y w*; *dTOR^ΔP^/+*; *fkh^6^/+*), indicating that FKH function is required for target gene induction by low TOR signaling. (**C**) The body weight of adult flies heterozygous for *dTOR^ΔP^* is increased by the presence of one copy of the *fkh^1^* allele. Both male and female transheterozygous flies are slightly but significantly heavier than *dTOR^ΔP^/+* flies. Significance was tested using an unpaired 2-tailed Student's t-test. * = p<0.05; ** = p<0.01; *** = p<0.001. Error bars represent SEM.

In an attempt to establish quantitative molecular readouts which may be used to monitor FKH activity under conditions of normal or impaired TOR signaling, we screened published transcriptomic datasets for *Drosophila* genes which are regulated by nutrient availability, rapamycin or forkhead transcription factors in several independent experiments. The gene *cabut* encodes a C2H2 zinc finger transcription factor which is conserved between species (the yeast ortholog is FZF1, human ortholog is KLF11), plays a role in JNK-dependent dorsal closure in the *Drosophila* embryo [22] and was previously identified in screens for genes involved in axon guidance and synaptogenesis [23] as well as autophagic cell death [24]. *cabut* caught our interest because it was found to be transcriptionally upregulated by rapamycin in S2 cells, and RNAi knockdown of the gene lead to increased cell size and proliferation [25]. It therefore is a promising candidate growth suppressor gene downstream of TOR complex 1. It was also identified as being transcriptionally repressed by a *hs-fkh* transgene in late prepupae [26] and strongly induced by sugar condition (protein deprivation) in feeding larvae, but not by complete starvation [27]. Expression of an active dFOXO mutant in S2 cells leads to a moderate induction of *cabut* expression, while refeeding in adult flies has a weak repressing effect [28]. The *CG6770* gene was first identified as a transcriptional target of starvation in *Drosophila* larvae, encoding a protein with vague similarity to the pancreatic P8 transcriptional regulator from mouse and human. Expression was induced by both sugar condition and complete starvation, similar to the dFOXO target gene *d4E-BP* [27]. In agreement with these results, *CG6770* was found to be transcriptionally repressed by refeeding after starvation in adult *Drosophila* flies and moderately induced by expression of activated dFOXO in S2 cells [28]. It was furthermore identified as a negative regulator of cell size in a genome-wide RNAi screen [29]. We first tested whether the transcription of *cabut* and *CG6770* is dependent on FKH levels. The driver lines used were the same as in the larval size experiments (Figure 1), that is *armadillo*-GAL4 for targeted RNAi and *pumpless*-GAL4 for ectopic expression. RNAi knockdown of FKH caused a significant decrease of *cabut* and *CG6770* mRNA in larval extracts, while FKH overexpression induced the transcription of both genes (Figure 5A). The induction of *CG6770* was several fold higher than that of *cabut*. We further validated the regulation of *CG6770* by FKH with a cell culture-based reporter gene experiment. When a 880 bp PCR product encompassing the *CG6770* promoter was cloned upstream of a luciferase ORF, reporter gene induction could be achieved from the resulting plasmid by co-expression of FKH to a greater extent than by dFOXO (Figure S3). We are aware of the fact that these observations do not exclude the possibility that *cabut* and *CG6770* are indirect FKH target genes, however we consider this of little relevance for their use as indicators for FKH activity. In a next step following the demonstration that both genes are induced by FKH, we addressed whether *cabut* and *CG6770* transcription was also responsive to TOR signaling levels, and could thus be used as a readout to confirm the interaction between the TOR module and FKH. Rapamycin feeding elicited a robust induction of *cabut* as well as *CG6770* expression in larvae (Figure 5B). Transcription of both genes was likewise elevated in heterozygous and homozygous *TOR* mutant larvae (Figure 6B). The highest levels of *CG6770* expression, which apparently is more sensitive to TOR signaling levels than *cabut* expression, were measured in rapamycin-fed larvae which also expressed transgenic FKH (Figure 5C), although the difference in *CG6770* transcript levels to the FKH-expressing larvae without rapamycin is not significant.

### FKH is required for the response to rapamycin and lowered TOR levels

Based on the observations described above, we addressed whether FKH plays a functional role of physiological relevance downstream of TOR. Several lines of evidence indicate that FKH is required for the induction of *cabut* and *CG6770* as well as for growth inhibition under conditions of low TOR signaling. First, target gene mRNA levels in FKH-RNAi larvae fed with rapamycin were lower than in wild-type or lacZ-RNAi control animals subjected to the same treatment (Figure 6A), indicating that FKH function is required for full expression of both genes upon TOR inhibition. However, both genes still appear to be induced by rapamycin in FKH-RNAi larvae, because FKH knockdown also reduces basal *cabut* and *CG6770* transcript levels in the absence of rapamycin. Second, larvae with reduced FKH levels were less susceptible to growth inhibition by rapamycin (Figure 1B and F). Likewise, individual cells in which FKH expression was silenced had a growth advantage over the neighboring wildtype cells under conditions of starvation or TOR inhibition (Figures 2 and 3). Third, the augmented transcription of *CG6770* and *cabut* in heterozygous *TOR* mutant larvae was reversed to wild-type levels in double mutant animals carrying a single copy of the *fkh^1^* or the *fkh^6^* loss-of-function allele [15] in addition to the *TOR* mutation (Figure 6B). Fourth, presence of the *fkh^1^* allele significantly increased the body weight of adult flies heterozygous for a *TOR* mutation (Figure 6C). Taken together, these results demonstrate that FKH function is required *in vivo* for the inhibition of organismal and cellular growth and the induction of target gene transcription under conditions of low TOR pathway activity.

### Impact of FKH and TOR on the expression of the dFOXO target *d4E-BP*

**Figure 7:**
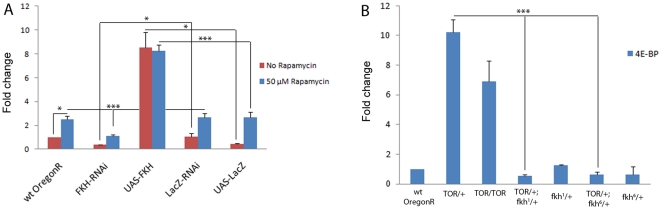
FKH and TOR impact on the expression of the dFOXO target *d4E-BP*. Realtime qPCR was applied to quantify mRNAs in larval extracts. (**A**) Compared to wildtype and unspecific control animals, transcript levels of *d4E-BP* are low in larvae expressing FKH dsRNA (*w; arm-Gal4/+*; *UAS-FKH-RNAi/+*) and high in larvae overexpressing FKH (*w;; ppl-Gal4/UAS-FKH*). *d4E-BP* is upregulated in larvae treated with rapamycin, and the elevated *d4E-BP* transcription resulting from rapamycin feeding is completely suppressed by FKH RNAi. (**B**) *d4E-BP* is upregulated in *dTOR* mutants (*y w; dTOR^ΔP^/+ and y w*; *dTOR^ΔP^/dTOR^ΔP^*) and the expression is suppressed in larvae transheterozygous for dTOR and FKH mutations (*y w; dTOR^ΔP^/+*; *fkh^1^/+ and y w*; *dTOR^ΔP^/+*; *fkh^6^/+*). This suggests that *d4E-BP* is a transcriptional target not only of the insulin pathway and dFOXO, but also of the TOR pathway and FKH. Significance was tested using an unpaired 2-tailed Student's t-test. * = p<0,05 ** = p<0,01 *** = p<0,001. Error bars represent SEM.

Finally, we investigated the convergence of transcriptional regulation downstream of the insulin and TOR signaling modules. More specifically, we measured the impact of FKH and TOR signaling on the expression of the dFOXO target gene *d4E-BP/Thor*. The O subfamily forkhead transcription factor dFOXO is negatively regulated by insulin signaling and activates transcription of *d4E-BP*, which in turn represses cap-dependent translation under conditions of nutrient scarcity [30], [31]. Rapamycin was found to induce transcription of *d4E-BP* in larvae, whereas RNAi-knockdown of FKH lead to a significant decrease of expression under both conditions. Conversely, overexpression of FKH elicited a strong elevation of *d4E-BP* mRNA levels (Figure 7A). Similar to the effect on *CG6770* and *cabut*, *TOR*-mutant larvae displayed increased *d4E-BP* transcription, which was completely suppressed by the *fkh^1^* or the *fkh^6^* mutation (Figure 7B). These results suggest that induction of *d4E-BP* transcription is not only mediated by dFOXO under conditions of starvation and low insulin signaling, but also by FKH when TOR signaling levels are low.

## Discussion

**Figure 8:**
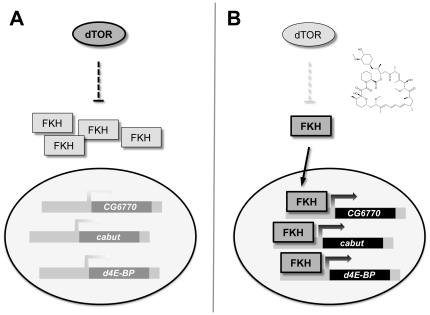
A simplified working model of nutrient-dependent gene expression by TOR and FKH. (**A**) Under conditions of dietary protein abundance, the TOR signaling module is active and exerts a negative regulation on FKH, which is consequently sequestered in the cytoplasm and unable to modulate gene transcription. (**B**) When TOR complex 1 activity is inhibited by rapamycin or protein deprivation, the repression of FKH activity is diminished. A significant fraction of the cellular FKH pool accumulates in the nucleus and activates expression of the growth-inhibiting genes *CG6770*, *cabut* and *d4E-BP*.

In *C. elegans*, the FoxA transcription factor PHA-4 has been identified as a mediator of TOR signaling in the regulation of lifespan. In this study, we present a characterization of the fly FoxA protein FKH in the biological context of TOR-regulated growth and gene transcription. Figure 8 shows a simplified schematic representation of the working model which we propose based on our experimental results, describing FKH as a mediator of the cellular response to protein deprivation.

As FKH has been previously uncharacterized in terms of growth control, we first established that alterations of FKH levels lead to changes in cellular and organismal size. Ectopic expression of FKH reduced growth of feeding larvae and of fatbody cells under conditions of nutrient abundance, and induced expression of the rapamycin-sensitive genes *CG6770* and *cabut*. The observation that FKH overexpression phenotypes are strongest under conditions of dietary protein abundance and active TOR signaling might reflect a scenario in which the abundance of upstream negative regulators of FKH is limiting under these conditions. Thus an experimental elevation of FKH protein levels would override the endogenous upstream signaling systems and elicit an inhibition of growth. As discussed below, the same argumentation could explain the finding that in contrast to the endogenous protein, overexpressed FKH accumulates in the nucleus even on a high-protein diet. The same three readouts were used to demonstrate that FKH function is required for growth inhibition downstream of TORC1. Targeted knockdown of FKH by RNAi diminished the decrease in larval size, fatbody cell size as well as the induction of rapamycin target gene expression when TOR signaling activity was lowered. The elevated transcription of *CG6770*, *cabut* and *d4E-BP* in heterozygous *TOR* mutants was likewise suppressed by *fkh* loss-of-function alleles, corroborating the findings obtained from our RNA interference experiments.

In addition to its function in the regulation of TOR-dependent growth and gene expression, we investigated the impact of nutrient levels and TOR signaling on the subcellular localization of FKH. On a protein-rich-diet, FKH was excluded from the nucleus in larval fatbody tissue, and inhibition of TOR signaling by pharmacological or genetic means elicited a robust nuclear translocation of the transcription factor. Starvation induced by complete nutrient deprivation on PBS only had a weak effect on FKH localization, diminishing nuclear exclusion to the point that the contours of the nuclei, which were visible due to the absence of nuclear signal in the yeast feeding condition, were no longer discernible. Albeit weak, we believe that this increase in nuclear localization is biologically relevant, as FKH function is required for the starvation-induced attenuation of cell growth in the larval fatbody (Figures 2 and 3). As described above, the subcellular distribution of FKH in starved animals is in stark contrast to the one of dFOXO, which is predominantly nuclear in this nutritional state. Another apparent difference between the shuttling of FKH and dFOXO is that in the case of dFOXO, both the overexpressed and endogenous protein translocates to the cytoplasm upon insulin stimulation of cells. This likewise holds true for the mammalian FOXO proteins. The situation for FOXA proteins appears to be somewhat less uniform. We report for the fly FOXA/FKH that the endogenous protein is excluded from the nuclei in the larval fatbody under conditions of highly abundant dietary protein and amino acids, which translates into high TOR signaling activity. In contrast, overexpressed FKH protein is constitutively nuclear even on a high protein diet. The cause for this may be that the abundance of one or several upstream regulators is limiting and tuned to endogenous FKH concentrations. If the copy number of FKH protein is elevated beyond physiological levels, it may escape negative regulation and nuclear export induced by the endogenous signaling components, and thus accumulate in the nuclei. In the field of mammalian forkhead transcription factors, the insulin-dependent nucleocytoplasmic shuttling of FOXO proteins is well established [3]. Also mouse Foxa2 has been shown to translocate to the cytoplasm under conditions of high insulin signaling in cultured cells as well as in tissues such as liver and hypothalamus [32], [33], [34], but there have been conflicting reports arguing that hepatic Foxa2 is constitutively nuclear, irrespective of metabolic state or insulin levels [35], [36]. It remains to be investigated whether the regulation of *Drosophila* FKH localization and activity by TORC1 described here is conserved in mammalian orthologs such as Foxa2. Conversely, it remains to be investigated whether the TOR-dependent nuclear exclusion of FKH is a consequence of protein phosphorylation. Cytoplasmic sequestration of mouse Foxa2 is linked to phosphorylation of T156 by AKT, a residue within a sequence motif that is conserved in *Drosophila* FKH [34]. In the light of our study and the interaction of FoxA/PHA-4 with TOR and S6K in *C. elegans* [11], it is also tempting to speculate that FoxA proteins may be regulated through phosphorylation by TOR or S6K.

*d4E-BP* has been previously described as a target of dFOXO [30], [31]. We have demonstrated that *d4E-BP* expression is silenced by low FKH levels and elevated upon FKH overexpression. Moreover, *4E-BP* transcript levels are elevated upon pharmacological or genetic inactivation of TOR. As with the FKH target genes *cabut* and *CG6770*, the positive effect of rapamycin or TOR mutation on *4E-BP* expression is suppressed by FKH knockdown or FKH loss-of-function mutations. We therefore argue that *d4E-BP* is most likely a common transcriptional target of both dFOXO and FKH and thus constitutes an additional point of crosstalk between the insulin and TOR signaling modules. The fact that FKH and dFOXO share the conserved forkhead DNA binding domain may suggest at least partially overlapping target gene populations. Regarding the growth-suppressive action of FKH under conditions of nutrient deprivation, it is currently unclear whether growth inhibition is mainly achieved through the induction of *CG6770*, *cabut* and *d4E-BP* or if other, yet unidentified FKH targets also contribute to this phenotype. *d4E-BP* mutants have no overgrowth phenotype under normal conditions [37], but it remains to be investigated whether they display impaired growth inhibition under starvation conditions. The observation that the *Thor^1^* allele partially suppresses the *Akt1* cell number phenotype in the eye provides evidence that d4E-BP function is required when insulin signaling levels are lowered [30]. In cultured cells, knockdown of *CG6770* [29] or *cabut* [25] leads to increased cell size, raising the possibility that they also act as negative regulators of growth downstream of FKH. Further studies are required to assess the relative relevance of the individual target genes in this context.

In summary, we present the first evidence that the interaction between TOR signaling and FoxA proteins is conserved in *Drosophila*. Our study supports a model in which FKH is regulated by the TOR pathway and dFOXO by the insulin/PI3K pathway, two signaling systems that are already interwoven at several points such as TSC2 and 4E-BP. On the level of transcription factors, there seems to be differential regulation: FKH is activated under conditions of protein deprivation and low TOR signaling, while dFOXO is activated by complete starvation and low insulin signaling. However, there is also a downstream node of convergence. The expression of the translational inhibitor d4E-BP, which has been established as a dFOXO target and is transcriptionally induced by protein deprivation as well as complete starvation [27], is induced under conditions of low TOR signaling by FKH. This emerging molecular scenario outlined by these observations would allow cells and organisms to react specifically to different conditions of nutrient availability and food composition.

## Materials and Methods

### Constructs, antibodies and fly lines

The open reading frames (ORFs) encoding FKH (a protein comprising 510 amino acid residues) and dFOXO (a protein comprising 613 amino acid residues) were PCR-amplified from genomic DNA (*fkh* is a single-exon gene) isolated from adult flies and from a pUAST-dFOXO plasmid template [30], respectively. The amplicons were cloned into pENTR/D-TOPO (Invitrogen) to generate gateway entry constructs, which were fully sequenced to ensure sequence integrity. Cell culture expression constructs were then created by transferring the ORFs via LR recombination into the modified gateway destination vector pAHW-Blast, which corresponds to the pAHW plasmid from Terence Murphy's *Drosophila* Gateway Vector Collection [38] with a blasticidin resistance cassette cloned into the backbone, and allows expression of N-terminally 3xHA-tagged proteins under control of the Actin5C promoter. Furthermore, a pAGW-FKH construct was generated to allow expression of a green fluorescent EGFP-FKH fusion protein. P-element based expression constructs for transgenic flies were created by transferring the *fkh* ORF into pTHW and pTRW from the same vector collection. The resulting plasmids pTHW-FKH and pTRW-FKH allow GAL4-induced expression of 3xHA-FKH and red fluorescent mRFP1-FKH, respectively. An inverted repeat construct for the targeted *in vivo* knockdown of FKH by RNAi was created by first PCR-amplifying the first 701 bp of the *fkh* ORF with a XhoI linker added to the forward and a BglII linker to the reverse primer. The resulting amplicon was digested with XhoI and then self-ligated with T4 DNA ligase. The correct 1.4 kb ligation product was gel purified, digested with BglII and cloned into the BglII site of pMF3 [39]. The cloning step was performed in SURE bacteria (Stratagene). The CG6770 reporter plasmid pGL3-CG6770 was constructed by cloning a 880 bp PCR amplicon covering the *CG6770* regulatory region up to the translation start codon into the pGL3-Basic vector containing the gene encoding *Photinus pyralis* (firefly) luciferase (Promega) as a BglII/blunt fragment. pGL3-Basic was first digested with NcoI, the resulting 5′ overhangs were then blunted with T4 DNA polymerase before proceeding with BglII digestion as the second step. The PCR was performed with a 5′-phosphorylated reverse primer to ensure efficient ligation by T4 DNA ligase. The control construct for expressing *Renilla reniformis* luciferase under control of the RpIII128 promoter, polIII-RL [40], was a kind gift of Norbert Perrimon. Phusion DNA polymerase (Finnzymes) was used for all PCR steps in this study. All primer sequences are provided as supporting information in Text S1. A polyclonal antibody against FKH was generated by Coring Systems Diagnostix (Gernsheim, D). αFKH1 was raised in rabbit against the peptide SHSSLEATSPGKKD, and purified by immunoaffinity chromatography. The location of the peptide in the FKH protein sequence is provided as supporting information in Text S3. Two independent transgenic UAS-FKH-RNAi lines were mainly used. One was established with our pMF3-FKH construct described above, and one was obtained from the Transgenic RNAi Project (TRiP) at Harvard Medical School (TRiP stock JF02417) [41]. A third construct which was generated by cloning a 913 bp amplicon spanning part of the *fkh* ORF and part of the *fkh* 3′-UTR into the Sym-pUAST-w vector [42] was found to be less effective in inducing *in vivo* knockdown compared to the other two constructs (data not shown), and was therefore not further used in most experiments. The regions in the *fkh* mRNA which are targeted by the three individual RNAi constructs are provided as supporting information in Text S2. All *in vivo* RNAi experiments which involved knockdown of FKH expression were performed in parallel with transgenics carrying the pMF3 construct or the TRiP construct, and identical phenotypes were observed for both. The loss-of-function allele *dTOR^ΔP^*, which is a deletion generated by imprecise P-element excision and removes the *dTOR* translation start site as well as the amino-terminal 902 codons [14], was a gift of Tom Neufeld. The *fkh* loss-of-function alleles *fkh^1^* (stock nr. 3331) and *fkh^6^* (stock nr. 545) were obtained from the Bloomington *Drosophila* Stock Center. *fkh^1^* contains an ethyl methanesulfonate (EMS)-induced point mutation changing the codon for W254, a residue in the forkhead DNA-binding domain, into a stop codon, while the X-ray-induced allele *fkh^6^* carries an 11 bp deletion which produces a frameshift after the codon for residue E7 [15]. FKH overexpression *in vivo* was achieved by use of transgenic UAS-3xHA-FKH or UAS-mRFP1-FKH lines which were established with our pTHW-FKH and pTRW-FKH constructs.

### Larval starvation and rapamycin feeding

If not stated otherwise, embryos were collected for 4 h on PBS-agar plates with yeast paste (prepared by suspending one cube of 42 g fresh yeast in 6.5 ml PBS). The fatbody samples shown in Figure 2 are derived from larvae that were treated as follows: for the “yeast” condition, larvae were kept on yeast paste until 64 h AED. For the “PBS” condition, larvae were kept on yeast paste until 64 h AED, washed out of the yeast paste, rinsed with water and transferred to a petri dish with PBS-soaked filter paper for another 24 h. For the “rapamycin” condition, larvae were kept on yeast paste until 48 h AED, before 200 µl of a 50 µM rapamycin (LC Laboratories) solution was added to the yeast paste and the plates were further incubated for 24 h at 25°C.

### Immunohistochemistry

Antibody stainings were performed as described before [43]. In brief, larval tissues were dissected in *Drosophila* Ringer's solution and fixed for 30′ in 4% formaldehyde in PBS+0.5% Tween-20 (PBT). Tissue was blocked with 5% Goat Serum in PBT for 30′, and washed before and after incubation with primary antibody in PBS+0,5% Tween-20 or PBS+0.1% Tween-20, respectively, for 5/5/15/30′. Incubation with primary antibodies diluted in blocking solution was performed over night at 4°C. Fluorescence-coupled secondary antibodies were applied for 1 h at RT. DAPI (1 µg/ml) was included in the last washing step before samples were mounted in Mowiol (Roth). Primary antibodies used were rabbit αFKH1 (dilution 1∶200), mouse anti-GFP (Sigma, dilution 1∶500), guinea pig anti-ppl (a gift of Ingo Zinke, dilution 1∶500) and mouse anti-CD2 (Serotec, dilution 1∶200). Secondary antibodies (all diluted 1∶200 in PBS+0.1% Tween-20+5% Goat Serum) used were Alexa Fluor 488 goat anti-mouse IgG and Alexa Fluor 546 goat anti-rabbit IgG (Invitrogen) and Cy5 anti-guinea pig (Jackson ImmunoResearch). Mounted tissue was analyzed using a Zeiss LSM 710 confocal microscope and images were further processed with the Zeiss LSM Image Software. The size of cell clones in fatbody samples was measured using the ImageJ software.

### Cell culture and luciferase assays

*Drosophila* S2R+ cells were obtained from the Drosophila Genomics Resource Center (DGRC stock nr. 150), cultured at 25°C in Schneider's *Drosophila* medium (Invitrogen) containing 10% heat-inactivated FCS (Sigma). Cells were split and diluted to a density of 1×10^6^ per ml once per week. For reporter gene assays, S2R+ cells were transfected with the FuGene HD reagent (Roche) in 24 well-plates (Greiner). Per well, 50 ng of the reporter construct pGL3-CG6770 and 5 ng polIII-RL were used. Where indicated, 200 ng of expression plasmids pAHW-FKH-Blast or pAHW-dFOXO-Blast were co-transfected with the reporter gene constructs. After the cells had grown in the multiwell plates to a density of ca. 80%, transfection was performed for 8 h, after which cells were allowed to recover on serum-containing medium for 12 h and subsequently subjected to serum deprivation over night. Cells were lysed in Passive Lysis Buffer before measuring firefly and *Renilla* luciferase activities subsequently in each sample according to the Dual Luciferase System protocol (Promega) in a Wallac luminometer (PerkinElmer). The firefly luciferase values were normalized to the *Renilla* values to account for variations in transient transfection efficiency.

### Realtime PCR

Quantitative realtime PCR to measure transcript levels was done as described previously [20]. Briefly, total RNA was extracted from *Drosophila* larvae with TriFast (peqlab)/chloroform in a Precellys homogenisator (peqlab), following the manufacturer's protocol. Extracted RNA was dissolved in DEPC-treated water. To minimize RNA degradation, RNA was stored at −80°C and used as a template for cDNA synthesis within 24 h. 1 µg of total RNA was used for cDNA synthesis with the QuantiTect kit (Qiagen). cDNA integrity and absence of contamination by genomic DNA was assessed by a PCR amplification of Actin5C from + and − reverse transcriptase reactions. Realtime qPCR was performed with a CFX96 instrument (Bio-Rad). Primers were designed with the GC-content recommended for qPCR (between 40% and 60%) and low self-complementarity using the software tool Primer3. Prior to quantitation experiments, primer efficiency and NTC (No Template Control) was tested for each primer pair to ensure an efficiency of at least 80% and rule out primer dimer formation. PCR reactions consisted of first-strand cDNA template and iQ SYBR Green Supermix (Biorad). Actin5C and rp49 transcript levels were used for normalization. All primer sequences are provided as supporting information in Text S1. For every mRNA quantitation, the minimal number of replicates consisted of biological triplicates, with each triplicate measured twice as technical replicates for a total of six measurements.

### Statistics

Error bars represent the standard error of the mean (SEM) if not stated otherwise. Statistical significance was assessed using an unpaired two-tailed Student's t-test, comparing the experimental data with the respective controls. For comparisons between one experimental value and two control values (such as wildtype and unspecific RNAi controls), analysis of variance (ANOVA) was used in addition to confirm the significance values derived from the pairwise t-tests. If not stated otherwise, all experiments were carried out at least twice independently. Asterisks indicate a p-value of <0.05 (*), <0.01 (**) or <0.001 (***). Significance was tested with the software InStat 3 from GraphPad Software.

## Supporting Information

Figure S1 Efficiency of RNAi-mediated knockdown. *fkh* mRNA levels were quantified in larval extracts by quantitative realtime PCR to control RNAi-mediated knockdown. Compared to wild-type larvae, expression of *fkh* dsRNA (pMF3-fkh construct) under control of the *armadillo* driver lead to a reduction of *fkh* transcript levels by 60%. Heterozygosity for the *fkh* ^1^ allele reduced larval transcript levels by 70% compared to wildtype animals.
(TIF)

Figure S2 Control of antibody specificity and localization of overexpressed FKH. FKH is the main protein recognized by the newly generated αFKH1 antibody in western blots and immunostainings, and overexpressed FKH is constitutively nuclear. (A) Western blot analysis of larval extracts probed with the αFKH1 antibody. Lane 1: in an extract from wild-type larvae, the antibody detects a single protein band of approximately 54 kDa, which is the predicted molecular weight of FKH and therefore most likely corresponds to endogenous FKH. Lane 2: in an extract from *w; UAS-mRFP1-FKH*; *ppl-GAL4* larvae, the 54 kDa protein is detected as well. In addition, a band of higher molecular weight is visible which corresponds to the transgenically encoded mRFP1-FKH fusion protein. (B–E) Immunofluorescent double staining of fatbody from *y w hs-FLP;; Act>CD2>Gal4 UAS-GFPnls/UAS-3xHA-FKH* larvae. The GFP-marked cell clones express transgenically encoded 3xHA-tagged FKH protein, which is recognized by a mouse monoclonal anti-HA antibody (B) as well as the rabbit polyclonal αFKH1 antibody (D). Secondary antibodies used were anti-mouse-Cy3 and anti-rabbit-Cy5. For both antibodies, one panel shows the only the signal of the actual immunostaining (B and D), panel C shows the merged signals of the clone marker GFP and and the nuclear DAPI stain, and panel E a merged picture with all four channels. Also when both stainings are performed separately on different batches of tissue from larvae of the indicated genotype, the nuclear 3xHA-FKH is detected by αFKH1 as well as anti-HA (data not shown). This demonstrates that αFKH1 is a suitable tool to visualize FKH in immunostainings, and that the main nuclear signal detected by the antibody corresponds to FKH protein and not an unspecific protein recognized by the antibody. In contrast to the endogenous protein (see figure 4), overexpressed FKH is localized in the nucleus also under conditions of high TOR and insulin signaling. The fatbody shown in panels B–E is from a population of larvae which had been reared on protein-rich yeast paste, nevertheless the overexpressed 3xHA-FKH protein is nuclear. We also investigated the subcellular localization of overexpressed fluorescent FKH fusion proteins in cultured cells. (F–H) In S2R+ cells that had been transiently co-transfected with pAct5C-GAL4 and pTRW-FKH and were growing in serum-containing medium, the red fluorescent mRFP1-FKH protein is localized in the cell nuclei. (I–K) Likewise, in S2R+ cells that had been transiently transfected with pAGW-FKH, were growing in serum-containing medium and had been furthermore stimulated with 100 nM bovine insulin for 20 min., the green fluorescent EGFP-FKH is nuclear. Panels F and I show the signal of the respective fluorescent FKH fusion proteins, panels G and J the DAPI DNA stainings of the same confocal sections, and H and K the merged pictures containing signals from both channels. The same nuclear localization was observed in cells that had either been treated with 20 nM rapamycin for 30 min. or serum-deprived over night and subsequently subjected to PI3K inhibition with 50 µM LY294002 for 1 h (data not shown).
(TIF)

Figure S3 Induction of CG6770 promoter activity by FKH. Over-expressed FKH protein activates transcription from the *CG6770* promoter in cultured cells. S2R+ cells were transiently tranfected with a reporter plasmid containing the firefly luciferase gene under control of the *CG6770* regulatory region. The *Renilla* luciferase construct polIII-RL was co-transfected as an internal control to compensate for well-to-well variation in transfection efficiency. Before lysis and luciferase measurements, cells were incubated in serum-free medium over night to lower growth factor signaling levels. Compared to cells transfected with the luciferase vectors only, co-transfection of the dFOXO expression plasmid pAHW-dFOXO-Blast lead to a several fold induction of luciferase expression from the CG6770 promoter. Expression of FKH by co-transfection with pAHW-FKH-Blast elicited a much stronger induction of the reporter construct, leading to luciferase levels that were 5 fold higher than in the dFOXO-expressing cells and 20 fold higher compared to the control cells without expression vector.
(TIF)

Figure S4 Correlation with TSC1/2 and Rheb gain-of-function phenotypes. Inhibition or activation of TOR signaling leads to similar phenotypes as FKH overexpression and knockdown, respectively. (A and C) On a protein-rich yeast paste diet, co-expression of TSC1 and TSC2 in cell clones in the larval fatbody leads to a strong reduction in cell size. (B) The growth-inhibiting effect of TSC1/2 expression is much less pronounced in starved animals. (D) Conversely, activation of TOR signaling by expression of the small GTPase Rheb (Saucedo *et al.*, 2003; Stocker *et al.*, 2003) has a very mild growth-promoting effect on a protein-rich diet and (E and F) a stronger one under conditions of starvation. The same driver line was used as in the experiments shown in figure 2. A similar correlation was observed when using the expression of FKH target gene candidates as a readout. (G) Like FKH knockdown, expression of Rheb (driven by *arm*-*Gal4*) silences transcription of *CG6770* and *d4E-BP*. Inhibition of TOR signaling by TSC1/2 expression (driven by *ppl*-*Gal4*) leads to a strong elevation of mRNA levels of both genes, as does FKH expression. These observations further strengthen our model that FKH is functionally linked to the TOR signaling module.
(TIF)

Text S1 Sequences of primers used in this study.
(PDF)

Text S2 Regions in the *fkh* mRNA sequence targeted by the individual RNAi constructs used.
(PDF)

Text S3 Location of the peptide used for antibody generation within the FKH protein sequence.
(PDF)
