# Supplementary figures and images for: The Drosophila FoxA Ortholog Fork Head Regulates Growth and Gene Expression Downstream of Target of Rapamycin
Source: PLoS One. 2010 Dec 31;5(12):e15171. doi: 10.1371/journal.pone.0015171 (PMC3013099; doi:10.1371/journal.pone.0015171)

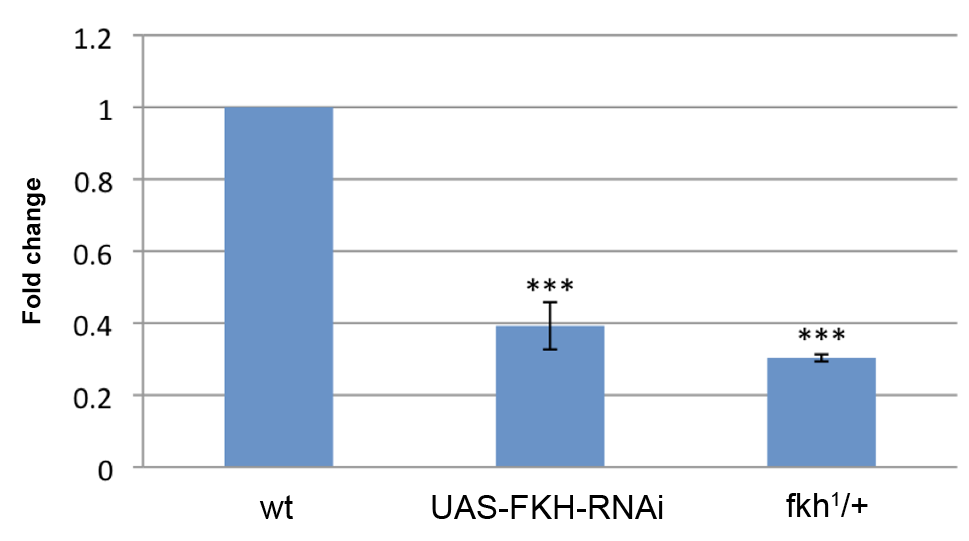

Supplement: Figure S1 — Efficiency of RNAi-mediated knockdown. fkh mRNA levels were quantified in larval extracts by quantitative realtime PCR to control RNAi-mediated knockdown. Compared to wild-type larvae, expression of fkh dsRNA (pMF3-fkh construct) under control of the armadillo driver lead to a reduction of fkh transcript levels by 60%. Heterozygosity for the fkh 1 allele reduced larval transcript levels by 70% compared to wildtype animals. (TIF) [file pone.0015171.s001.tif]

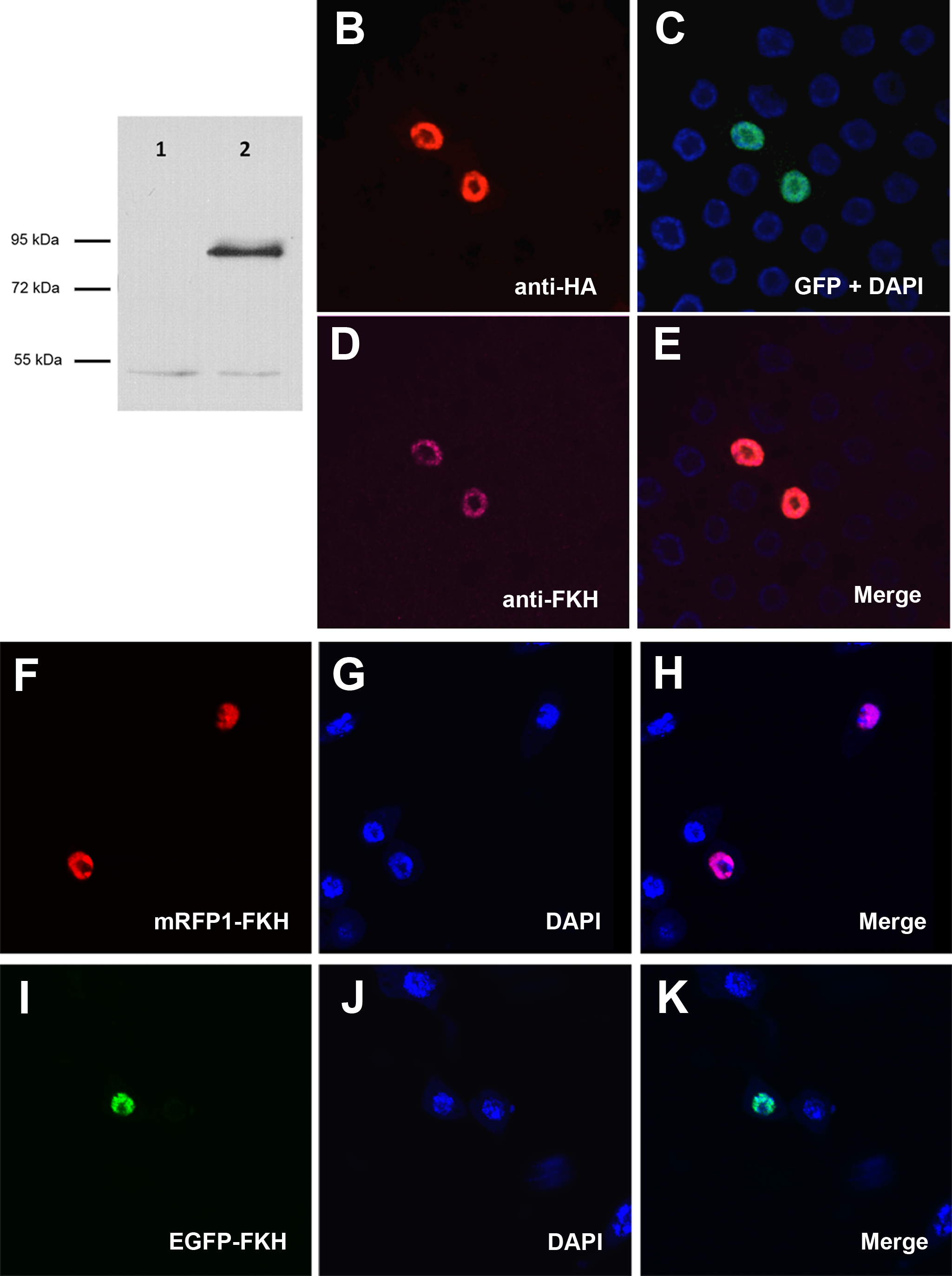

Supplement: Figure S2 — Control of antibody specificity and localization of overexpressed FKH. FKH is the main protein recognized by the newly generated αFKH1 antibody in western blots and immunostainings, and overexpressed FKH is constitutively nuclear. (A) Western blot analysis of larval extracts probed with the αFKH1 antibody. Lane 1: in an extract from wild-type larvae, the antibody detects a single protein band of approximately 54 kDa, which is the predicted molecular weight of FKH and therefore most likely corresponds to endogenous FKH. Lane 2: in an extract from w; UAS-mRFP1-FKH; ppl-GAL4 larvae, the 54 kDa protein is detected as well. In addition, a band of higher molecular weight is visible which corresponds to the transgenically encoded mRFP1-FKH fusion protein. (B–E) Immunofluorescent double staining of fatbody from y w hs-FLP;; Act>CD2>Gal4 UAS-GFPnls/UAS-3xHA-FKH larvae. The GFP-marked cell clones express transgenically encoded 3xHA-tagged FKH protein, which is recognized by a mouse monoclonal anti-HA antibody (B) as well as the rabbit polyclonal αFKH1 antibody (D). Secondary antibodies used were anti-mouse-Cy3 and anti-rabbit-Cy5. For both antibodies, one panel shows the only the signal of the actual immunostaining (B and D), panel C shows the merged signals of the clone marker GFP and and the nuclear DAPI stain, and panel E a merged picture with all four channels. Also when both stainings are performed separately on different batches of tissue from larvae of the indicated genotype, the nuclear 3xHA-FKH is detected by αFKH1 as well as anti-HA (data not shown). This demonstrates that αFKH1 is a suitable tool to visualize FKH in immunostainings, and that the main nuclear signal detected by the antibody corresponds to FKH protein and not an unspecific protein recognized by the antibody. In contrast to the endogenous protein (see figure 4), overexpressed FKH is localized in the nucleus also under conditions of high TOR and insulin signaling. The fatbody shown in panels B–E is fro [file pone.0015171.s002.tif]

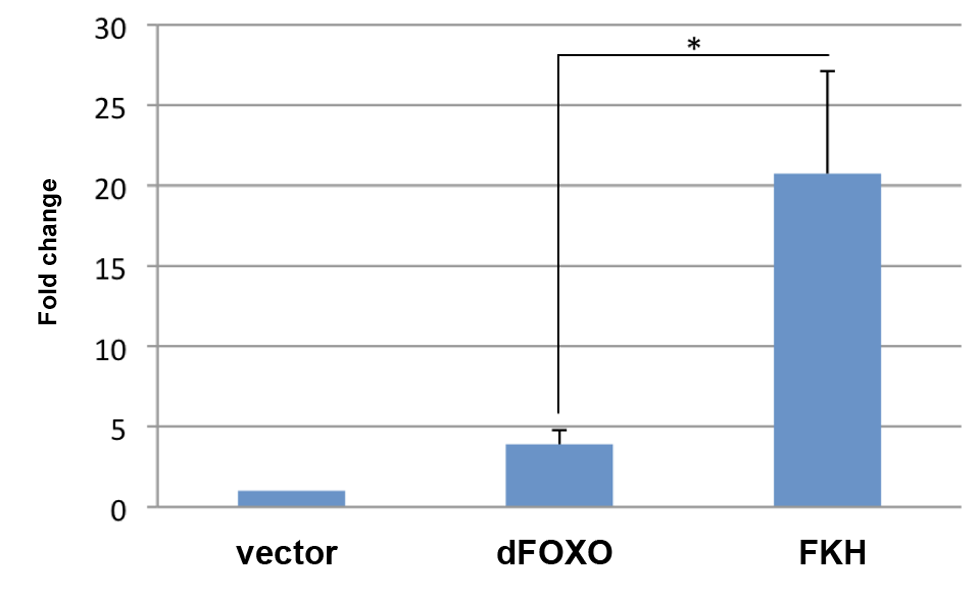

Supplement: Figure S3 — Induction of CG6770 promoter activity by FKH. Over-expressed FKH protein activates transcription from the CG6770 promoter in cultured cells. S2R+ cells were transiently tranfected with a reporter plasmid containing the firefly luciferase gene under control of the CG6770 regulatory region. The Renilla luciferase construct polIII-RL was co-transfected as an internal control to compensate for well-to-well variation in transfection efficiency. Before lysis and luciferase measurements, cells were incubated in serum-free medium over night to lower growth factor signaling levels. Compared to cells transfected with the luciferase vectors only, co-transfection of the dFOXO expression plasmid pAHW-dFOXO-Blast lead to a several fold induction of luciferase expression from the CG6770 promoter. Expression of FKH by co-transfection with pAHW-FKH-Blast elicited a much stronger induction of the reporter construct, leading to luciferase levels that were 5 fold higher than in the dFOXO-expressing cells and 20 fold higher compared to the control cells without expression vector. (TIF) [file pone.0015171.s003.tif]

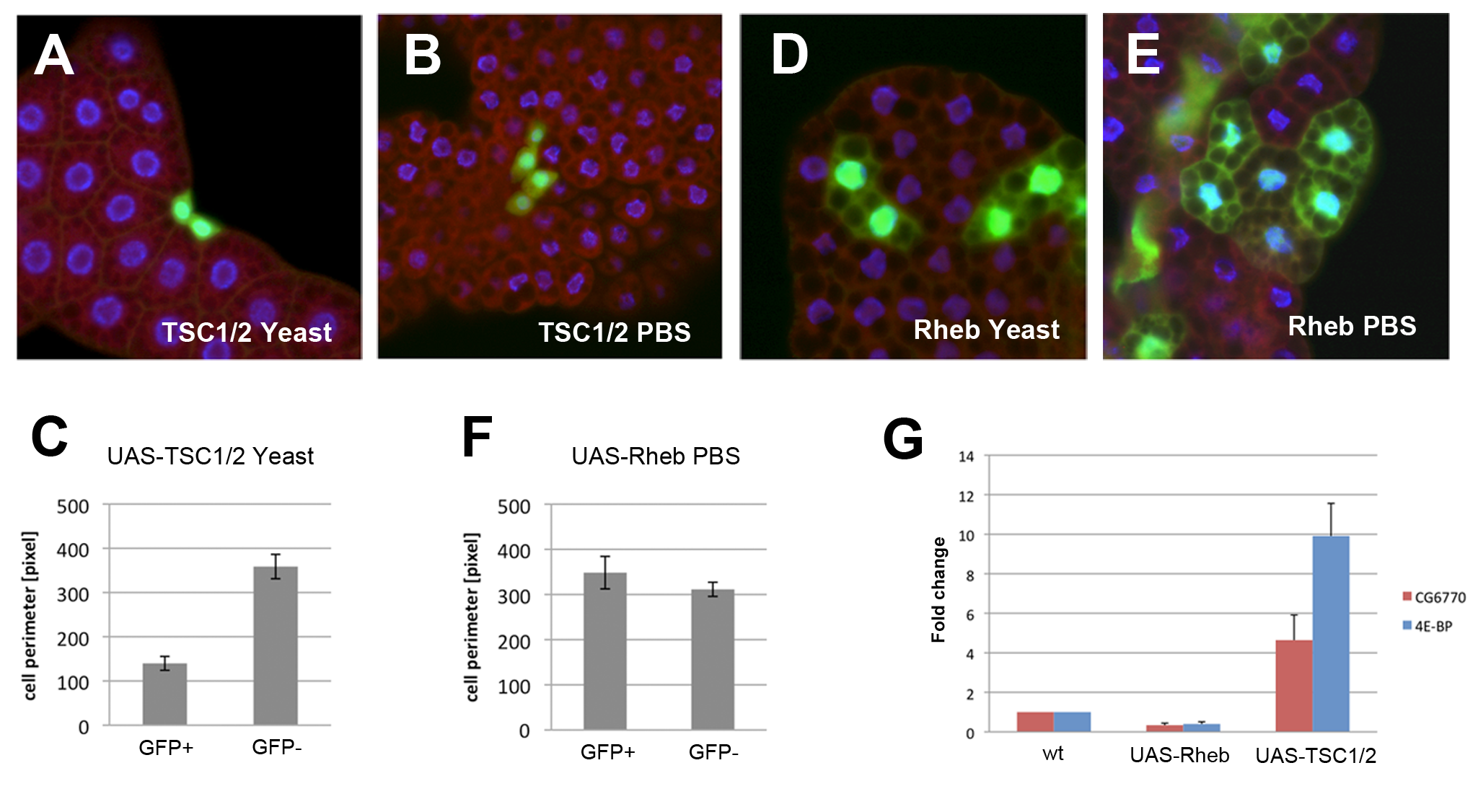

Supplement: Figure S4 — Correlation with TSC1/2 and Rheb gain-of-function phenotypes. Inhibition or activation of TOR signaling leads to similar phenotypes as FKH overexpression and knockdown, respectively. (A and C) On a protein-rich yeast paste diet, co-expression of TSC1 and TSC2 in cell clones in the larval fatbody leads to a strong reduction in cell size. (B) The growth-inhibiting effect of TSC1/2 expression is much less pronounced in starved animals. (D) Conversely, activation of TOR signaling by expression of the small GTPase Rheb (Saucedo et al., 2003; Stocker et al., 2003) has a very mild growth-promoting effect on a protein-rich diet and (E and F) a stronger one under conditions of starvation. The same driver line was used as in the experiments shown in figure 2. A similar correlation was observed when using the expression of FKH target gene candidates as a readout. (G) Like FKH knockdown, expression of Rheb (driven by arm-Gal4) silences transcription of CG6770 and d4E-BP. Inhibition of TOR signaling by TSC1/2 expression (driven by ppl-Gal4) leads to a strong elevation of mRNA levels of both genes, as does FKH expression. These observations further strengthen our model that FKH is functionally linked to the TOR signaling module. (TIF) [file pone.0015171.s004.tif]
