# Supplementary material for: The Drosophila FoxA Ortholog Fork Head Regulates Growth and Gene Expression Downstream of Target of Rapamycin
Source: PLoS One. 2010 Dec 31;5(12):e15171. doi: 10.1371/journal.pone.0015171 (PMC3013099; doi:10.1371/journal.pone.0015171)
Supplement: Text S1 — Sequences of primers used in this study. (PDF) [file pone.0015171.s005.pdf]

Supporting information: Text S1

Primer sequences

PCRs to generate FKH and dFOXO gateway entry clones

FKH fwd: CACCATGCAGAAGCTCTACGCGGAGCCGC
FKH rev: CAAGCTCGTGGTTCCGGCGGGC

dFOXO fwd: CACCATGATGGACGGCTACGCGCAGGAATGG
dFOXO rev: GTGCACCCAGGATGGTGGCGAGG

PCR product for pMF3-FKH construct

FKH IR fwd: AATACCCTCGAGATGCAGAAGCTCTACGCGGAGCCG
FKH IR rev: AATACCAGATCTGAGAGCGTCAACATTCTGGTGGGG

PCR product for pGL3-CG6770 construct

CG6770 fwd: ATACCAGATCTCAAATGCGTTGCCTGGTGGGAGGG
CG6770 rev: ATGGCGATTTAAGGTTGCAAAACTAACTGCGTTCTTGGTTGG

Realtime PCR primers

Actin5C fwd: GATCTGGCTGGTCGCGATT
Actin5C rev: GGCCATCTCCTGCTCAAAGTC

rp49 fwd: TCCTACCAGCTTCAAGATGAC
rp49 rev: CACCTTGTGCACCAGGAACT

CG6770 fwd: TCGACCATGACAAGCACATC
CG6770 rev: GGCCCCGTTACTTCTTTCTC

cabut fwd: ACAACAAGGACAAGGCGAAC
cabut rev: GAACTGATGGGCGAGGAAC

d4E-BP fwd: GAAGATTGAGGACCAGGAACA
d4E-BP rev: CGAGAGAACAAACAAGGTGGA

DInR fwd: CACCCCGCTTCTATACTCCA
DInR rev: GTTAGGATGGTGGCCTGTG
