# Supplementary material for: The Drosophila FoxA Ortholog Fork Head Regulates Growth and Gene Expression Downstream of Target of Rapamycin
Source: PLoS One. 2010 Dec 31;5(12):e15171. doi: 10.1371/journal.pone.0015171 (PMC3013099; doi:10.1371/journal.pone.0015171)
Supplement: Text S2 — Regions in the fkh mRNA sequence targeted by the individual RNAi constructs used. (PDF) [file pone.0015171.s006.pdf]

Supporting information: Text S2

Different RNAi amplicons used for *in vivo* FKH knockdown

Dark Blue/**Dark Red**: pMF3-FKH construct
Dark Red: part overlapping between pMF3-FKH and TRiP construct
Dark Red/**Red**/**Light Blue**: TRiP construct JF02417
Light Blue: part overlapping between TRiP and Sym-pUAST-w-FKH construct
Light Blue/**Green**: Sym-pUAST-w-FKH construct

FKH mRNA sequence (UTRs in lowercase):

agtggccaacagccagctagatattcacgttcgtgcgtcggtgttttgtg
gccgctcggtgcggcagacaacaaaaatccccatacaaaaatacaaagaa
aatacaaaaagaatcgaatcataacagaaaataaatatatatttatatg
attatcccgcacaaataattaaaccacggtgtgtgtttacaaacaaagct
tagaatcagaatcagaatcgcaaacggaatcagaaccttccagccaacaa
gtgacaagtgccgaagtgctctcgataaaatgccagatatatgcaaaccg
attgaagcagtgatagtgcactgaaatcacagttgatatacaatatatca
gagtaaagccacgttttaaagacctcctttttgattttcgttttaactaa
cgcgttgcagaacgcgttgccttttccatcgatccaccagagggcagcgc
ctccgaaagccggcaacataacggcgcaactattgaacagctttctgaag
ggtagcccccaccagaccaccattgacatc**ATGCAGAAGCTCTACGCGGA**
GCCGCCTCCGAGCAGTGCTCCGGTGAGCATGGCCAGCTCGGGTGGCGGTG
GTCCTCCGTCGGGCGGCGGTGGAGGCGGCGGCGGAGGAGGAGGCGGTGGG
CCCCCACCGCCCAGCAATAACAACCCGAATCCGACGAGCAACGGCGGCAG
CATGAGTCCCCTGGCTCGATCCGCCTACACAATGAACAGCATGGGCCTGC
CGGTGGGTGGCATGTCATCGGTTTCCCCTCAGGCGGCGGCCACATTCAGC
TCCAGCGTCCTGGACTCGGCGGCGGCGGTCGCCAGCATGAGCGCCAGCAT
GAGTGCCAGTATGAGCGCCAGCATGAATGCCAGCATGAACGGCAGCATGG
GTGCGGCGGCCATGAACTCAATGGGCGGCAACTGCATGACCCCCAGCTCG
ATGAGCTACGCCAGCATGGGATCACCGCTCGGGAACATGGGTGGCTGCAT
GGCCATGTCGGCAGCGAGTATGTCGGCGGCGGGATTGAGCGGCACCTATG
GCGCCATGCCGCCGGGATCCCGGGAAATGGAGACGGGATCGCCGAATTCC
CTAGGCAGATCGCGGGTGGACAAACCAACTACGTACAGAAGGAGCTACAC
GCATGCCAAGCCGCCATACAGCTACATCTCACTGATCACCATGGCCATTC
AGAATAACCCCACCAGAATGTTGACGCTCTCGGAGATCTATCAGTTCATC
ATGGATCTGTTTCCGTTCTACAGGCAGAATCAGCAGCGCTGGCAGAACTC
CATTCGACATTCGCTGAGCTTCAACGATTGCTTCGTAAAGATTCCCAGGA
CGCCGGACAAGCCAGGAAAGGGATCGTTTTGGACTCTGCATCCGGATTCG
GGGAATATGTTCGAGAACGGGTGCTATTTGCGGCGACAGAAGCGTTTCAA
GGACGAGAAGAAGGAGGCCATTAGGCAGCTGCACAAGAGTCCGTCGCACA
GCAGCCTGGAGGCCACCAGTCCGGGCAAGAAGGATCACGAGGACTCGCAC
CACATGCACCACCACCACCACAGCCGGCTGGACCATCATCAGCACCACAA
GGAGGCGGGCGGAGCATCGATCGCCGGCGTAAATGTCCTGAGTGCAGCGC
ACAGCAAGGACGCGGAGGCCCTGGCCATGTTGCATGCCAATGCCGAACTG
TGCCTCAGCCAGCAGCCGCAGCATGTGCCCACACATCACCACCATCAGCA
CCACCAGTTGCAGCAGGAGGAGCTGTCGGCGATGATGGCCAATCGGTGCC

ATCCGTCGCTGATCACCGACTACCACTCGTCGATGCATCCGCTGAAGCAG
GAGCCCTCTGGCTACACGCCCTCCAGCCATCCGTTCTCCATCAACCGCCT
GCTGCCCACGGAGTCCAAGGCGGACATCAAGATGTACGACATGAGCCAGT
ACGCCGGCTACAACGCCCTCAGTCCGCTGACCAACTCACACGCTGCCTTA
GGCCAGGACTCCTACTACCAGAGTCTTGGCTACCATGCGCCCGCCGGAAC
CACGAGCTTGTGAcatcaccaatacccgctggcttaaggacgcgcggagc
agatgctgcgctcgcagcagcaacagcacttgcagcagcagcaccagcaa
caccagcagcaacagcagcagcaccagctgcatcagcagcagcagcagat
gcagcagtcggcgcagcaactgacatccgcttccaatacaccagcaacat
cagcaaaggccagcggcaaggcgggatcggcttcggcctcgggatctggg
tcaggatcgggttcaggatcgggctctaactactcacagaagctgcaaca
gcagcaccagcagcagcaacaacagcaggcggcggcgcagcagcaacacc
accagcagcagcaacaactgctgcaggaactgcaggaggactcatcgaac
atcaccagcgatctgagcgaggagcaactgcagcagcatcaggccgccca
gcagcagttgtacaacaactatcagcagtatgccgcggcggcaggctaca
gaaattggaatcacggcctggctttcaatggagccgccgctctgcagaat
cttctgtagctatagctatagatcccgatctccacaggcagcggctttgt
agcgggcctgccaattagcgagtgcagtggcggtggcttttagcacctgg
ccgcatccgcatccgcactcgcatcctcatcctttgagcacgtgagtctt
gggccgcaactcgcacttgtacatatagagatacctatgaagagccgatc
gagagcgctgccttttgtgttgttcagttccagtactatatattgtagat
tatatagattctgttccgggggttattaaatcgacatagagcaggagga
ggaggagcgaaagaaaccgtgatgtagttgtaagtgcagcagaactttg
ttaacaattttacgttttgctaatacaaatacaaaataataattccgtgc
catttgttttcgttgtttcgaaacaagcaataacaatcaaagcggttata
actgaaaatttgaaatcacattttaaatgtatccgattaaggagaagatt
gaggagaggagatgtaaagaagggatcgtgtgtaatgttctttcataaaa
caaagccccaaatgaaacagtcccaaaaagagagatacaaatactaaacc
ctttatccaaaggcaatcgcaatcgaattcgaatcgcaaaagatcaaaac
caaaaagaaatctaaaac
