# Supplementary material for: The Drosophila FoxA Ortholog Fork Head Regulates Growth and Gene Expression Downstream of Target of Rapamycin
Source: PLoS One. 2010 Dec 31;5(12):e15171. doi: 10.1371/journal.pone.0015171 (PMC3013099; doi:10.1371/journal.pone.0015171)
Supplement: Text S3 — Location of the peptide used for antibody generation within the FKH protein sequence. (PDF) [file pone.0015171.s007.pdf]

Supporting information: Text S3

FKH peptide used for antibody generation: location in FKH protein sequence

Red: Peptide SHSSLEATSPGKKD
Blue: Forkhead DNA-binding domain

MQKLYAEPPPSSAPVSMASSGGGGPPSGGGGGGGGGGGGGPPPPSNNNPN
PTSNGGSMSPLARSAYTMNSMGLPVGGMSSVSPQAAATFSSSVLDSAAAV
ASMSASMSASMSASMNASMNGSMGAAAMNSMGGNCMTPSSMSYASMGSPL
GNMGGCMAMSAASMSAAGLSGTYGAMPPGSREMETGSPNSLGRSRVDKPT
TYRRSYTHAKPPYSYISLITMAIQNNPTRMLTLSEIYQFIMDLFPFYRQN
QQRWQNSIRHSLSFNDCFVKIPRTPDKPGKGSFWTLHPDSGNMFENGCYL
RRQKRFKDEKKEAIRQLHKSP**SHSSLEATSPGKKD**HEDSHHMHHHHHSRL
DHHQHHKEAGGASIAGVNVLSAAHSKDAEALAMLHANAELCLSQQPQHVP
THHHHQHHQLQQEELSAMMANRCHPSLITDYHSSMHPLKQEPSGYTPSSH
PFSINRLLPTESKADIKMYDMSQYAGYNALSPLTNSHAALGQDSYYQSLG
YHAPAGTTSL
